# Supplementary material for: Rapid turnover of life-cycle-related genes in the brown algae
Source: Genome Biol. 2019 Feb 14;20:35. doi: 10.1186/s13059-019-1630-6 (PMC6374913; doi:10.1186/s13059-019-1630-6)
Supplement: Supplementary file 3 — Protein alignments, corresponding Gblocks html file and CDS sequences for the lineage conserved gametophyte-biased genes. (ZIP 2109 kb) [file 13059_2019_1630_MOESM3_ESM.zip › Con_GA_alignments/Ec-01_004600.aa_ali.fasta-gb.htm]

Ec-01\_004600.aa.fasta.trans.aa\_ali.fasta


## Gblocks 0.91b Results

Processed file: **Ec-01\_004600.aa.fasta.trans.aa\_ali.fasta**  
Number of sequences: **2**  
Alignment assumed to be: **Protein**  
New number of positions: **301** (selected positions are underlined in blue)

```
                         10        20        30        40        50        60
                 =========+=========+=========+=========+=========+=========+
Ec-01_004600     MSFPDTRPIS--AQGLEVTMKAAEDEARSTQR--HHQQVVGDCASTTTTTGATAVHAGSQ
MSTRG.47416.1    MSNFGMRPISTLDQEAERATAAQEEEATASKARENQRQVVGNSATTSRSDEATPQGSIQH
                 ##########   ##############          ################       


                         70        80        90       100       110       120
                 =========+=========+=========+=========+=========+=========+
Ec-01_004600     GQQQQQQLVGVDAKTNLHAQQQQTHGKGPGAARDEGAEKDAEGNGDGDEACGEDTHLGAR
MSTRG.47416.1    HHHHH-----------NQVQPPQSHGTVGGSINQDVGDNVAAQEKEDDAGSREDAHLGAH
                                    ###########          ####################


                        130       140       150       160       170       180
                 =========+=========+=========+=========+=========+=========+
Ec-01_004600     APDLRPWSRMEDEAIARMVVERGIKRWSVIAGNLYVEVAGTLRTGKQCKARWQHMLDPSQ
MSTRG.47416.1    TPDQQPWSSREDEAITRMVLEGGIKRWSVIAGNLFAEVAGTLRTGKQCRARWLQVLEPSL
                 ############################################################


                        190       200       210       220       230       240
                 =========+=========+=========+=========+=========+=========+
Ec-01_004600     KNAPWTEEEENIIHDAHLRLGNRWSEIAKLLTGRTDSAVKNYWYTTAHKNIGRQNMEIR-
MSTRG.47416.1    KESSWTTEEEQTFNDAQLRLGSNWAEIAKLLNGRTEDAVKKHWYASVHKNIHRQQMDGGD
                 ########################################################    


                        250       260       270       280       290       300
                 =========+=========+=========+=========+=========+=========+
Ec-01_004600     -----GGDGDGRSDPSRSGDTEATGSSNINPIGPGGESVATN-------GGASGFYDDPK
MSTRG.47416.1    TTVATTAKEEGESGTGGLKSSGEDGSSYHGLVGKSGSLTVVPRCFGGNVGGSASGFGAPK
                                         ############             ###########


                        310       320       330       340       350       360
                 =========+=========+=========+=========+=========+=========+
Ec-01_004600     PDIVPGEAGIAGGCSAPVGHVEGGECQLQPVVAGLGVCGGIGPPPAMAVATAAAVATHGQ
MSTRG.47416.1    PNTLRGQVEIPDPYSISVGHVGGVEGLLPPVVAGLGVCEGMGRSFPPAVATSSV---MGQ
                 ###################################################       ##


                        370       380       390       400       410       420
                 =========+=========+=========+=========+=========+=========+
Ec-01_004600     GMPPVGAATAAPGVESSSPPALVPSL---------------------------ASTL---
MSTRG.47416.1    GAAPVARTQLAMKAESSSSPALGPVIALSAMVDGREHWQASEDQKRGFFNQPRAGSLAKT
                 ########################                                    


                        430       440       450       460       470       480
                 =========+=========+=========+=========+=========+=========+
Ec-01_004600     --------GTTAPPAVDGNNRWCPTTTSGV------------------------------
MSTRG.47416.1    QTDIENNAREQFNKQVDGNIRGASGSSSGITGNPTDRVAAATVGLQHGNRFFPRESSSGQ
                                ##############                               


                        490       500       510       520       530       540
                 =========+=========+=========+=========+=========+=========+
Ec-01_004600     ------------------------------------------------------------
MSTRG.47416.1    AAGEASSSLVLGQAVVDGRMASGVPTKSPPLPAPASSVGSRGCDQHVKVGAKRARPRLFV
                                                                             


                        550       560       570       580       590       600
                 =========+=========+=========+=========+=========+=========+
Ec-01_004600     ------------------------------------------------------------
MSTRG.47416.1    DTNVDATIRHGGHEPHRHFVQHQQHRPVDQHQHQPGFETKGMNASATAAVAAAPSSLVRA
                                                                             


                        610       620       630       640       650       660
                 =========+=========+=========+=========+=========+=========+
Ec-01_004600     ------------------------------------------------------------
MSTRG.47416.1    SNAVSASAAPYGSPPGVGSSAAEAASKAAEAAARYASGLVSAGNLCLPSAHFHHAQQHQQ
                                                                             


                 
                 ===
Ec-01_004600     ---
MSTRG.47416.1    HQQ
```

```
Parameters used
Minimum Number Of Sequences For A Conserved Position: 2
Minimum Number Of Sequences For A Flanking Position: 2
Maximum Number Of Contiguous Nonconserved Positions: 8
Minimum Length Of A Block: 10
Allowed Gap Positions: None
Use Similarity Matrices: Yes
```

```
Flank positions of the 9 selected block(s)
Flanks: [1  10]  [14  27]  [38  53]  [80  90]  [101  236]  [265  276]  [290  351]  [359  384]  [436  449]  

New number of positions in Ec-01_004600.aa.fasta.trans.aa_ali.fasta-gb:  301  (45% of the original 663 positions)
```
